# Effect of One Percent Chlorhexidine Addition on the Antibacterial Activity and Mechanical Properties of Sealants: An *in vitro* Study

**DOI:** 10.5005/jp-journals-10005-1312

**Published:** 2015-09-11

**Authors:** Arunachalam Karthikeyan Shanmugaavel, Sharath Asokan, J Baby John, PR Geetha Priya, Jagadeesan Gnana Devi

**Affiliations:** Postgraduate Student, Department of Pedodontics and Preventive Dentistry, KSR Institute of Dental Science and Research, Tiruchengode, Tamil Nadu, India; Professor, Department of Pediatric Dentistry, KSR Institute of Dental Science and Research, Tiruchengode, Tamil Nadu, India; Professor and Head, Department of Pediatric Dentistry, KSR Institute of Dental Science and Research, Tiruchengode, Tamil Nadu, India; Reader, Department of Pediatric Dentistry, KSR Institute of Dental Science and Research, Tiruchengode, Tamil Nadu, India; Dental Surgeon, Department of Dentistry, Arunz Dental Clinic, Chennai, Tamil Nadu, India

**Keywords:** Antibacterial activity, Chlorhexidine, Pit and fissure sealants.

## Abstract

**Aim:** The aim of the study was to evaluate the effect of addition of 1% chlorhexidine digluconate solution on the antibacterial activity and mechanical properties of glass ionomer and resin based sealant.

**Materials and methods:** Conventional glass ionomer sealant (GIS) (Fuji VII, Japan) and resin sealant (Clinpro 3M ESPE, USA) were used in this study. Chlorhexidine digluconate (CHX) (20%) liquid was added to both the sealants, and the concentration of chlorhexidine in sealants was adjusted to 1%. The sealants were divided into four groups as: group A (GIS), group B (GIS + 1% CHX), group C (resin sealant), group D (resin sealant + 1% CHX). Five cylindrical specimens were prepared in each group. Their antibacterial activity against *Streptococcus mutans* and *Lactobacillus acidophilus,* and their mechanical properties (compressive strength and diametrical tensile strength) were assessed. Mann-Whitney and Wilcoxon signed rank test were used appropriately for statistical analysis (SPSS version 19).

**Result:** Addition of one percent chlorhexidine significantly increased the antibacterial activity of both the sealants. There was a significant difference between groups A and B (p < 0.009), and groups C and D (p < 0.008). There was no significant difference in the mechanical properties of the sealants.

**Conclusion:** Addition of one percent chlorhexidine to the glass ionomer and resin based sealants provided sufficient antibacterial activity, without significantly affecting the mechanical property of the sealants.

**How to cite this article:** Shanmugaavel AK, Asokan S, John JB, Geetha Priya PR, Gnana Devi J. Effect of one percent Chlorhexidine Addition on the Antibacterial Activity and Mechanical Properties of Sealants: An *in vitro* Study. Int J Clin Pediatr Dent 2015;8(3):196-201.

## INTRODUCTION

‘An ounce of prevention is better than a pound of cure-Benjamin Franklin‘. Dental caries, second to common cold, remains one of the most prevalent chronic diseases that affect children and young adults globally. The world is now aiming to prevent this disease at an early stage and provide children the best quality of life. Pit and fissure sealants provide both primary and secondary levels of prevention by preventing caries initiation and progression.^1^ Microleakage around the sealants provide pathway for the bacteria and initiate secondary caries. If these caries lesions progress, they may eventually result in pain and pulp exposure in young children.

An antibacterial agent is a substance that either kills bacteria or inhibits their growth. Chlorhexidine diglu-conate is a cationic biguanide which has gained attention for its antibacterial properties. Jedrychowski (1983) was the first to report the antibacterial property of the restorative material after the addition of chlorhexidine.^2^ Many studies have been carried out by adding chlorhexidine to glass ionomer cement, but there were limited studies stating the addition of chlorhexidine to resin materials.^2-6^ Adding antibacterial activity to the sealants strengthens the defense mechanism of the later against the bacteria. Hence, this study was planned to evaluate the antibacterial activity and mechanical properties of the glass ionomer and resin based sealants after the addition of 1% chlorhexidine digluconate.

## MATERIALS AND METHODS

The study protocol was analyzed and approved by institutional review board of KSR institute of dental science and research, Tiruchengode, India. Conventional glass ionomer sealants (GIS) (Fuji VII, Japan) and resin sealants (Clinpro 3M ESPE) were used in this study. Test specimens were prepared by adding 20% chlorhexidine digluconate (CHX) liquid (Anabond Stedman pharma research, Chennai, India) in the following proportions: 0.05 ml of the CHX was pipetted and added to 0.95 ml of the liquid portion of the GIS and to the 0.95 ml of resin sealant to adjust the concentration to 1% CHX (w/w). The sealants were divided into four groups namely: group A (GIS), group B (GIS + 1% CHX), group C (resin sealant), group D (resin sealant + 1% CHX). Five specimens were prepared in each group. The antibacterial activity was assessed via agar diffusion test against *Streptococcus mutans* and *Lactobacillus acidophilus* in the department of biotechnology (KSR group of institutions, Thiruchen-gode, Tamil Nadu, India), and the mechanical properties (compressive strength and diametrical tensile strength) were examined at central institute of plastic engineering and technology, Chennai.

### Agar Diffusion Test

*Streptococcus mutans* and *L. acidophilus* are the most common pathogens involved in the initiation and progression of the carious lesion. These bacteria were obtained from microbial type culture collection and gene bank, Chandigarh, India. The antibacterial activity of the set material against *S. mutans* (MTCC 497; microbial type cell culture, Chandigarh) and *L. acidophilus* (MTCC 10307; microbial type cell culture, Chandigarh) was assessed using agar diffusion test. All the procedures were done in an aseptic laminar cabinet.

The strains of *L. acidophilus* and *S. mutans,* stored in 50% glycerol at -20°C were cultivated in brain heart infusion (BHI) and *Lactobacillus* MRS agar (Hi Media *Laboratories* Pvt. Ltd, Mumbai, India) broth, respectively at 37°C. A loopful of inoculum was transferred to 10 ml of BHI and *Lactobacillus* MRS agar broths after 48 hours incubation. Five wells of 6 mm diameter were cut using a well cutter in commercially available Muller Hinton agar (MHA) and Mullen Hinton agar supplied with 5% sheep blood (Bioline laboratory, Coimbatore, India) agar plates of 90 mm diameter and 4 mm thickness. Bacterial suspensions of 350 μl of *L. acidophilus* and *S. mutans* were streaked over the agar plates.

The GIS were mixed according to powder/liquid ratio of 1:1 with sterile spatula on a mixing pad and placed into wells within 1 minute after mixing. Similarly, the resin based sealants were placed into the wells and cured with halogen light curing unit (Elspar 2500, 3MESPE, USA) with light intensity of 410 mW/cm^2^, on both sides to ensure complete curing.^4^ Plain 20% CHX digluconate was used as positive control (E). The agar plates were kept at room temperature for 2 hours for diffusion of the material into the medium and incubated at 37°C for 48 hours. Zones of inhibition at day 0 were measured by a single calibrated examiner. The zones of inhibition were measured by subtracting the well diameter from the average zone of inhibition measured at three different points using digital vernier calipers (Aerospace, China) in millimeters (mm). The specimens were incubated for another 5 days at 37°C and then fresh subcultures were made; the procedure was repeated to measure the zones of inhibition after the 7th day. The antibacterial activity was measured again after 30 days by transferring the specimens at 23rd day and incubating for 48 hours in fresh inoculated plates.^4^

### Mechanical Property

Plastic molds were prepared according to American dental association (ADA) no. 27, of diameter 4 mm and height 6 mm. The GIS sealants were mixed as mentioned above and placed into a cylindrical plastic mold and covered with glass plates.^4^ The resin sealants were placed in the plastic mold in 2 mm increment for three times and cured with halogen light curing unit (Elspar 2500, 3MESPE, USA) with light intensity of 410 mW/cm^2^ on both sides to ensure complete curing. The materials were allowed to set for 30 minutes. The plastic molds were then removed and checked for the presence of voids. Specimens with voids were discarded. The cylindrical specimens were finished using carborundum disks and stored in distilled water for 24 hours.^4^ Prior to testing, the diameter of the specimens were determined using a micrometer gauge.

The specimens were then subjected to universal testing machine (Shimadzu, Japan). Compressive strength (CS) of the material was tested by giving load along the long axis of the specimen with cross head speed of 1 mm/min until fracture of the specimen occurs.^4^ The value of the force (F) measured in Newtons and diameter (d) was applied in the formula CS = (4F)/(πd^2^), to find out the compressive strength value in Megapascal (MPa).

Diametrical tensile strength (DTS) was measured by directing the force against the lateral surface of the specimen with cross head speed of 1 mm/min until fracture occurs.^4^ The value of the force (F) measured in Newtons and diameter (D) was applied in the formula DTS= (2F)/(7πDT), to find out the DTS value in Mega pascal (MPa).

The results were tabulated and statistically analyzed using SPSS version 19.0 software (SPSS Inc., Chicago Ill, USA). Mann-Whitney U test and Wilcoxon signed rank test were used appropriately for intergroup and intra-group comparisons. A p-value ≤ 0.05 was considered statistically significant.

## RESULTS

### Agar Diffusion Test

**Figs 1A to F:**
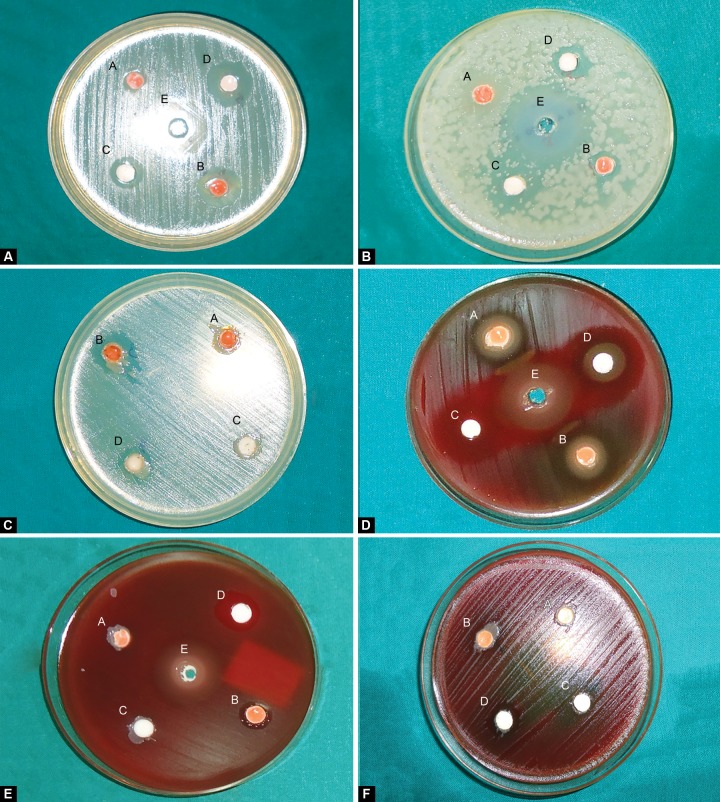
(A to C) Zones of inhibition in various groups against *L. acidophilus* on day 0, 7, 30 and (D to F) Zones of inhibition in various groups against S. *mutans* on day 0, 7, 30 (A: Glass ionomer sealant; B: GIS + 1% CHX; C: Resin sealant; D: Resin sealant + 1% CHX; E: 20% CHX)

Table 1 shows the results of agar diffusion test against *S. mutans* and *L. acidophilus* after 0, 7 and 30 days. Figures 1A to F depict the zones of inhibition for *L. acidophilus* and *S. mutans.* There was a significant increase in the antibacterial activity of the sealants against *S. mutans* [group B (p < 0.009); group D (p < 0.008)] and *L. acidophilus* [group B (p < 0.009); group D (p < 0.008)] immediately (day 0) after the addition of 1% chlorhexidine. This antibacterial activity decreased statistically after 7 days against *S. mutans* [group B (p < 0.042), group D (p < 0.042)] and *L. acidophilus* [group B (p < 0.041)]; while it was not statistically significant (NS) in group D (p < 0.068). This antibacterial activity continued to exist until 30 days. But no antibacterial activity was seen in conventional GIS and resin sealants (groups A and C) after 7 and 30 days.

**Table:** **Table 1:** Comparison of the mean zones of inhibition (mm) against S. *mutans* and *L. acidophilus* among the four groups at different time periods

		S. *mutans*							*L. acidophilus*					
*Groups*		*0 day*		*7 days*		*30 days*			*0 day*		*7 days*		*30 days*	
A		7.28		0		0			4.16		0		0	
B		10.36		5.7		1.67			9.7		5.02		1.5	
C		11.96		0		0			4.4		0		0	
D		14.82		8.68		5.83			10.18		8.16		4.83	

### Mechanical Property

Table 2 shows that the compressive and diametrical tensile strengths of the sealants were not altered significantly after the addition of 1% chlorhexidine.

## DISCUSSION

By any standards, advancements toward caries prevention have been impressive during the past few decades. Pit and fissures are eight times more susceptible than smooth surface to caries.^7^ Any tooth judged for caries risk can certainly benefit from sealant application, which acts as a physical barrier to microorganism and other food particles. Achieving a perfect isolation in young children is a very tough task. Placement of sealants is a very sensitive technique and is influenced by several factors, such as patient cooperation, contamination of the operating field and operator variability.^8^ The penetration of sealants in pits and fissures also depends on their.^9^ These factors can lead to microleakage, plaque accumulation around restorations or loss of restoration, which results in secondary caries. Secondary caries has two types of lesion namely: (A) outer lesion which occurs as a result of plaque accumulation, and (B) inner lesion which occurs due to microleakage between the tooth and restoration. Micro-leakage occurs around the sealants irrespective of the preparation methods and provides a pathway for the food particles, oral fluids and microorganisms to penetrate in the gaps, and initiate secondary caries.^10^ An ideal system with antibacterial activity reduces plaque accumulation on or near surfaces, and the number of microorganisms in oral cavity, thereby eliminating secondary caries.^3^

**Table:** **Table 2:** Comparison of the mechanical properties among the four groups

		*Compressive* * strength*				*Diametrical tensile* * strength*			
*Groups*		Mean ± SD* (MPa)†		*p-value*		Mean ± SD* (MPa)†		*p-value*	
A		17.10 ± 9.07		0.95 NS		6.5 ± 1.32		0.52 NS	
B		16.82 ± 4.63				5.51 ± 1.57			
C		123.54 ± 47.68		0.60 NS		43.37 ± 13.77		0.58 NS	
D		107.93 ± 42.06				32.69 ± 21.76			

*SD: Standard deviation; †MPa: Megapascal; NS: Nonsignificant

Chlorhexidine, a cationic polyguanide has been primarily used as disinfectant. The bactericidal effect is the result of this cationic molecule binding to the negatively charged bacterial cell walls. It is bacteriostatic and bactericidal at low and high concentrations respectively.

McCue et al (1951) have reported that quick setting acrylic has slight antibacterial activity.^11^ Its addition in mouthwashes has reduced plaque accumulation and hence helped in the maintenance of good oral hygiene. Since 1980, CHX addition has been tried in various forms and percentages in glass ionomer cement and composite resin to provide antibacterial activity. Chlorhexidine can be added to restorative materials either as powder or liquid, in percentages ranging from 1 to 10%.^2-5^ Jedry-chowski et al (1983) suggested that chlorhexidine added as powder form can be a better option than the liquid form.^2^ The antibacterial activity increased proportionately as the concentration of the liquid CHX increased but the mechanical properties were altered significantly in concentrations greater than 1%.^2,4^ Hence, in this study 1°% chlorhexidine digluconate liquid was added to the sealants. After CHX addition to resin sealants, it was stirred well with a sterile glass stirrer and the test specimens were prepared immediately. Agar plate diffusion was used in this study because it allowed analyzing both set and unset materials.^12^ This study was an attempt to add chlorhexidine to the sealants to enhance their antibacterial activity and make them more effective in prevention.

The differences in the zones of inhibition for *L. acido-philus* and *S. mutans* at three different time intervals are shown in Graphs 1A and 1B respectively. Results after day 0 showed antibacterial activity in all the groups, while no antibacterial activity was observed in groups A and C after 7 days and 30 days. This was in accordance with results shown by Naoungroj et al (2010) that conventional sealants provide surface antibacterial activity as a result of fluoride release.^13^ Matalon et al (2010) showed that sealants aged 30 days showed no antibacterial activ-ity.^14^ Specimens in groups B and D showed antibacterial activity even after a period of 30 days. Similar results were shown by Turkun et al (2008) in their study with antibacterial activity lasting for 70 days in GIC with 1.25% chlorhexidine digluconate.^4^ Takemura et al (1983) reported that the release of chlorhexidine from composite resin reduced greatly after 5 days immersion in water, although continued slow release was observed for 20 days.^6^

**Graph 1A:**
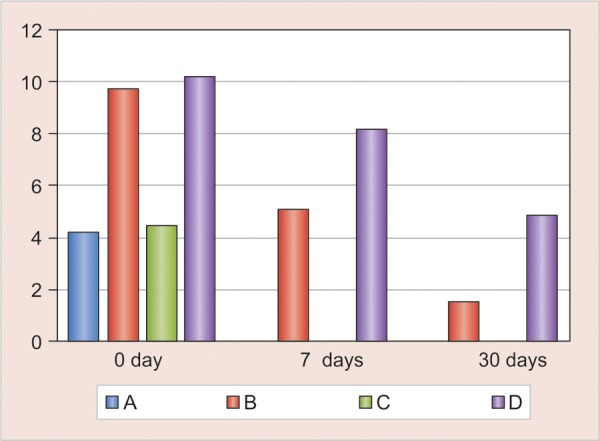
Comparison of mean scores of zones of inhibition of various groups against *L. acidophilus* on day 0,7,30

**Graph 1B:**
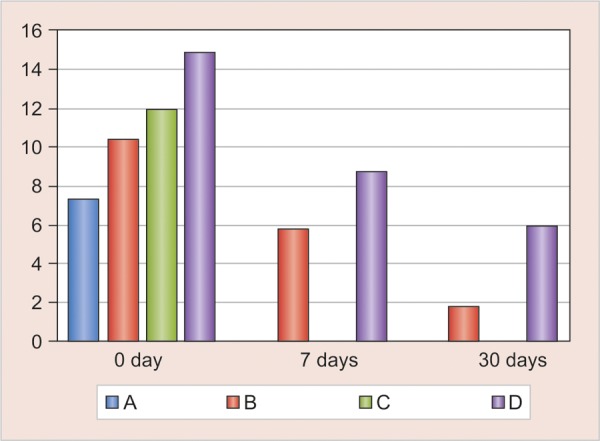
Comparison of mean scores of zones of inhibition of various groups against S. *mutans* on day 0,7,30; (A: Glass ionomer sealant; B: GIS + 1% CHX; C: Resin sealant, D: Resin sealant + 1% CHX)

The sealants showed reduction in the compressive and DTS after addition of 1% chlorhexidine digluconate, which is not statistically significant. It is in accordance to the results.^2-4^ The reduction in the mechanical properties may be due to the following reasons: (A) incorporated CHX interfered with the binding of the filler and matrix phase, (B) the CHX caused disturbance in the curing of monomers, and (C) release of the CHX could have caused a porous structure of the material.^15,16^ For the above reasons, Imatzo et al (2003) had suggested that addition of CHX was not appropriate for permanent restorations.^15^ Further studies are needed to evaluate the long-term effects of chlorhexidine addition on the mechanical properties of the sealants.

De Castilho et al (2013) investigated the *in vitro* and *in vivo* effects of addition of 1.25% CHX to resin modified glass ionomer (RMGIC) cement. *In vitro* results revealed an improved antibacterial property without affecting the mechanical property of the cement. *In vivo* results after 3 months showed complete elimination of *S. mutans* from the cavities restored with RMGIC with 1.25% chlorhexidine.^5^

The limitations of the current study include-a small sample size, the antibacterial activity was tested only for duration of 30 days, long-term effect on the mechanical properties of the sealants was not assessed, and chlor-hexidine added as a powder could have yielded better results. Further *in vivo* studies are needed to prove the clinical efficacy of chlorhexidine addition to sealants.

Within the limitations of this pilot study it can be concluded that addition of 1% chlorhexidine to the glass ionomer and resin based sealants provided additional antibacterial activity to the sealant without significantly affecting the mechanical property of the sealant. Antibacterial property of the sealants lasting for 30 days makes the sealants more effective in preventing dental caries, thereby improving the quality of life of young children.

## CONCLUSION

The addition of chlorhexidine in small concentrations provided antibacterial activity which can last for a period of 30 days without significantly altering the mechanical properties of the sealants. This can help the pediatric dentist give the child a better protection against caries and a promising healthy oral cavity.
